# Accuracy of Red Blood Cell Parameters in Predicting α^0^-Thalassemia Trait Among Non-Anemic Males

**DOI:** 10.3390/jcm14103591

**Published:** 2025-05-21

**Authors:** Benchaya Phanthong, Pimlak Charoenkwan, Threebhorn Kamlungkuea, Suchaya Luewan, Theera Tongsong

**Affiliations:** 1 Department of Obstetrics and Gynecology, Faculty of Medicine, Chiang Mai University, Chiang Mai 50200, Thailand; benchaya.p@cmu.ac.th (B.P.); threebhorn.k@cmu.ac.th (T.K.); 2 Department of Pediatrics, Faculty of Medicine, Chiang Mai University, Chiang Mai 50200, Thailand; pimlak.c@cmu.ac.th; 3 Thalassemia and Hematology Center, Faculty of Medicine, Chiang Mai University, Chiang Mai 50200, Thailand

**Keywords:** α^0^-thalassemia, hemoglobin, mean corpuscular volume, mean corpuscular hemoglobin, mean corpuscular hemoglobin concentration, red cell distribution width

## Abstract

**Background/Objectives**: Red blood cell (RBC) parameters are routinely used to screen for α- and β-thalassemia traits as part of prenatal diagnosis for severe fetal thalassemia in countries with a high prevalence of the disease. In clinical practice, the same cut-off values for these parameters are applied to both females and males. However, given that the normal reference ranges for some RBC parameters differ significantly between sexes, sex-specific cut-off values may be more appropriate, especially in combination. To date, the effectiveness of RBC indices in males for predicting α- and β-thalassemia traits has not been evaluated. The objectives of this study are to assess the diagnostic performance of individual and combined RBC parameters in detecting α^0^-thalassemia traits among non-anemic males. **Methods**: This diagnostic study is a secondary analysis of prospectively collected data from our project on prenatal control of severe thalassemia. The study population comprised male partners of pregnant women who underwent thalassemia screening during their first antenatal visit. RBC parameters, including hemoglobin (Hb), hematocrit (Hct), mean corpuscular volume (MCV), mean corpuscular hemoglobin (MCH), mean corpuscular hemoglobin concentration (MCHC), red cell distribution width (RDW), and RBC count, were measured for each participant. Carrier status for the α0-thalassemia Southeast Asian (SEA) genotype was confirmed by using a polymerase chain reaction (PCR)-based method. The diagnostic performance of each RBC parameter and their combinations, based on predictive models generated using logistic regression, was evaluated and compared using receiver operating characteristic (ROC) curves. **Results**: A total of 486 Thai males were recruited for the study, including 137 individuals with the α^0^-thalassemia trait and 349 with a normal α-thalassemia genotype (control group). All RBC parameters, except for Hct, differed significantly between the two groups. Among the individual indices, MCH exhibited the highest diagnostic accuracy, followed by MCV, with areas under the curve (AUCs) of 0.981 and 0.973, respectively. An MCH cut-off value of 26 pg and an MCV cut-off value of 80 fL provided a sensitivity of 100% for both indices, with specificities of 88.5% and 86.8%, respectively. The combination predictive model provided the best diagnostic performance, achieving an AUC of 0.987, which was slightly but significantly higher than that of any individual parameter. This model yielded a sensitivity of 100% and a significantly higher specificity of 90.8% at a cut-off probability of 7.0%. **Conclusions**: MCH and MCV demonstrated excellent screening performance for identifying α0-thalassemia carriers in males. However, the combination model exhibited even greater accuracy while reducing the false-positive rate. Implementing this model could minimize the need for unnecessary PCR testing, leading to substantial cost savings.

## 1. Introduction

Alpha-thalassemia is one of the most common genetic diseases worldwide [1,2], although its prevalence varies geographically, with the highest rates observed in Southeast Asia and China [3,4,5]. However, due to increased global migration in recent years, the prevalence has been rising in other regions, particularly in developed countries [4,6]. The disease is characterized by the underproduction of alpha-globin chains due to mutations of α-globin genes in the α-globin gene cluster located on the short arm of chromosome 16 [7]. Normal individuals possess four α-globin genes (αα/αα), with two alleles on each chromosome. In individuals with α-thalassemia, deletion of an α-globin gene results in reduced α-globin chain production and, consequently, in the case of deletion involving three or all four alleles, anemia [8]. The severity of α-thalassemia can be categorized into four groups: the silent carrier (–α/αα), also known as α^+^-thalassemia; the α-thalassemia trait, including homozygous α^+^-thalassemia (–α/–α) and heterozygous α^0^-thalassemia (– –/αα); hemoglobin (Hb) H disease (– –/–α); and homozygous α^0^-thalassemia (Hb Bart’s disease) (– –/– –). In Hb Bart’s disease, the absence of α-globin chain production leads to an excess of γ-globin chains and the formation of γ_4_ tetramers (Hb Bart’s). This disease is fatal, invariably leading to hydrops fetalis and severe maternal morbidity [7,8]. Hb Bart’s disease is highly prevalent in our country due to the high carrier rate of α^0^-thalassemia (Southeast Asian type; SEA), estimated at approximately 10% [2,9,10].

Due to its severity and high prevalence, prenatal screening and diagnosis for α0-thalassemia have been well established in clinical practice. The most common screening method for α0-thalassemia carriers is the determination of mean corpuscular volume (MCV), giving a sensitivity and specificity of 92.9% and 83.9%, respectively, at an MCV cut-off value of ≤80 fL [11]. Likewise, mean corpuscular hemoglobin (MCH) has also been shown to be an effective screening parameter for thalassemia, giving a sensitivity of 95.2% and a specificity of 82.3%, at a cut-off of <26.5 pg [12]. In practice, a positive screening result is further tested for confirmation by polymerase chain reaction (PCR), specifically targeting the SEA mutation, which is the most prevalent in our population [13]. A recent study has demonstrated that, in pregnant women, all common RBC parameters (Hb, MCV, MCH, MCHC, RDW, and RBC count) contribute to the prediction of α^0^-thalassemia carrier status. Moreover, a combination predictive model has proven highly effective in detecting α0-thalassemia carriers with a low false-positive rate, outperforming any single parameter [14]. The combined use of these parameters does not incur additional costs or require extra effort beyond that of using an individual parameter, as all values are derived from the same automated hematology analyzer. Therefore, leveraging all available RBC parameters in thalassemia screening is advantageous. Nevertheless, in clinical practice, identical cut-off values for determining a positive result are applied to both women and their male partners. While using the same cut-off for single parameters with minimal sex-related variation, such as MCV or MCH, may be of limited consequence, other parameters—particularly Hb levels and RBC counts—are consistently higher in males compared to females across diverse geographical populations [15,16,17,18]. Therefore, although combination models outperform individual parameters in diagnostic performance, the development of sex-specific models holds promise for further enhancing diagnostic accuracy. Therefore, this study aimed to assess the diagnostic performance of individual and combined RBC parameters in detecting α0-thalassemia traits among non-anemic males.

## 2. Patients and Methods

This study was conducted at a medical teaching hospital (tertiary center) within the Department of Obstetrics and Gynecology, Chiang Mai University, Thailand. The study design was diagnostic research involving a secondary analysis of prospectively collected data from our project on prenatal control of severe thalassemia, conducted between July 2017 and December 2022. Ethical approval for this study was obtained from the institutional review board (Research Ethics Committee 5, Faculty of Medicine, Chiang Mai University; research ID: OBG-2567-0735).

During the development of the primary project database, pregnant women and their partners were screened for thalassemia carrier status at their first antenatal care visit at our hospital and affiliated network hospitals in northern Thailand. The screening aimed to identify pregnancies at risk of carrying a fetus with severe thalassemia. All participants provided written informed consent before enrollment. Typically, both partners underwent initial screening using mean corpuscular volume (MCV). However, in this project, blood samples were analyzed for red blood cell (RBC) parameters, including hemoglobin (Hb), MCV, mean corpuscular hemoglobin (MCH), mean corpuscular hemoglobin concentration (MCHC), red cell distribution width (RDW), and RBC count. Carrier status for α^0^-thalassemia Southeast Asian (SEA) genotype was confirmed by using a polymerase chain reaction (PCR)-based method. Additionally, molecular diagnosis for α^+^-thalassemia mutations and hemoglobin typing to determine Hb A_2_ levels for the diagnosis of the β-thalassemia trait and Hb E trait were performed.

For database assessment, records of male partners meeting the following inclusion criteria were retrieved: (1) absence of underlying medical disorders, (2) no anemia, defined as an Hb level greater than 12 g/dL, (3) availability of hematologic data, including RBC parameters (Hb, hematocrit [Hct], MCV, MCH, MCHC, RDW, and RBC count), and (4) definitive diagnosis of thalassemia status based on molecular analysis of α^0^- and α^+^-thalassemia mutations and hemoglobin typing. Participants were excluded if they had anemia from any cause, such as iron deficiency anemia, chronic inflammatory disease, and thalassemia disease, or were carriers of other types of thalassemia, including the α^+^-thalassemia, β-thalassemia trait, or Hb E trait, which was routinely screened with the Hb E screen test [19].

The laboratory tests used in this study were as follows. Each blood sample (4 mL), collected at the antenatal care clinic, was analyzed for RBC parameters, hemoglobin typing, and molecular diagnosis of α^0^- and α^+^-thalassemia mutations. RBC parameters were measured using an automated hematology analyzer (Sysmex XN-9000; Sysmex Corporation, Kobe, Japan). Hemoglobin typing was performed using high-performance liquid chromatography (HPLC) with the Variant II HPLC system (Bio-Rad Laboratories, Berkeley, CA, USA). Molecular analysis for alpha-thalassemia, including α^0^-thalassemia (SEA and THAI deletions) and α^+^-thalassemia (3.7 and 4.2 kb deletions), was conducted using a gap-PCR method.

**Statistical analysis:** The statistical procedure was conducted using the Statistical Package for the Social Sciences (SPSS) software, version 26.0 (IBM Corp., 2019; IBM SPSS Statistics for Windows, Version 26.0, IBM Corp., Armonk, NY, USA). Continuous variables, such as red blood cell parameters, were presented as means ± standard deviation (SD), and comparisons between the normal and α^0^-thalassemia groups were performed using Student’s *t*-test. Binary logistic regression analysis was conducted using RBC parameters as predictors to develop a model for identifying α^0^-thalassemia carriers, incorporating the following variables: Hb, MCV, MCH, MCHC, RDW, and RBC count. The diagnostic performance of each parameter and the predictive model was evaluated using receiver operating characteristic (ROC) curves, and their performance was compared based on the area under the curves. Additionally, sensitivity and specificity at optimal cut-off values were determined using cross-tabulation analysis. To determine the optimal cut-off for defining an abnormal result, we prioritized achieving maximal sensitivity to meet the primary objective of α^0^-thalassemia screening. Rather than relying solely on statistical criteria, we deliberately chose a cut-off that maximizes sensitivity while maintaining an acceptable false-positive rate, recognizing that the overarching goal of screening is to minimize the risk of missing any true positive cases. A *p*-value of <0.05 was considered statistically significant.

## 3. Results

A total of 486 Thai non-anemic males who met the inclusion criteria were included in the study, comprising 137 (28.2%) males with the α^0^-thalassemia trait and 349 (71.8%) non-carrier controls. The mean (±SD) age of the participants was 30.36 ± 6.95 years, 30.14 ± 6.99 in the normal controls, and 30.92 ± 6.80 in the carrier group (Student’s *t*-test; *p*-value: 0.268). All other demographic characteristics did not differ significantly between the two groups, as detailed in Table 1.

Most red blood cell (RBC) parameters, except for Hct, differed significantly between α^0^-thalassemia carriers and the control group, as presented in Table 2. Males with the α^0^-thalassemia trait had significantly lower levels of Hb, MCV, MCH, and MCHC compared to the control group. In contrast, the RDW and RBC counts were significantly higher in α^0^-thalassemia carriers than in non-carrier individuals.

In constructing the predictive model, the resulting equation provides the log odds of being an α^0^-thalassemia carrier as follows:

Log (Odds of α^0^-thalassemia carrier) = 58.995 + 0.121 (Hb) − 0.200 (MCV) − 0.211 (MCH) − 1.319 (MCHC) − 0.280 (RDW) + 0.816 (RBC count)

where: Hb, hemoglobin; Hct, hematocrit; MCV, mean corpuscular volume; MCH, mean corpuscular hemoglobin; MCHC, mean corpuscular hemoglobin concentration; RDW, red blood cell distribution width; RBC counts, red blood cell counts.

The probability derived from this model approaches 1 in individuals at higher risk of carrying the α^0^-thalassemia trait, whereas a probability value approaching 0 indicates a lower risk.

**Figure 1:**
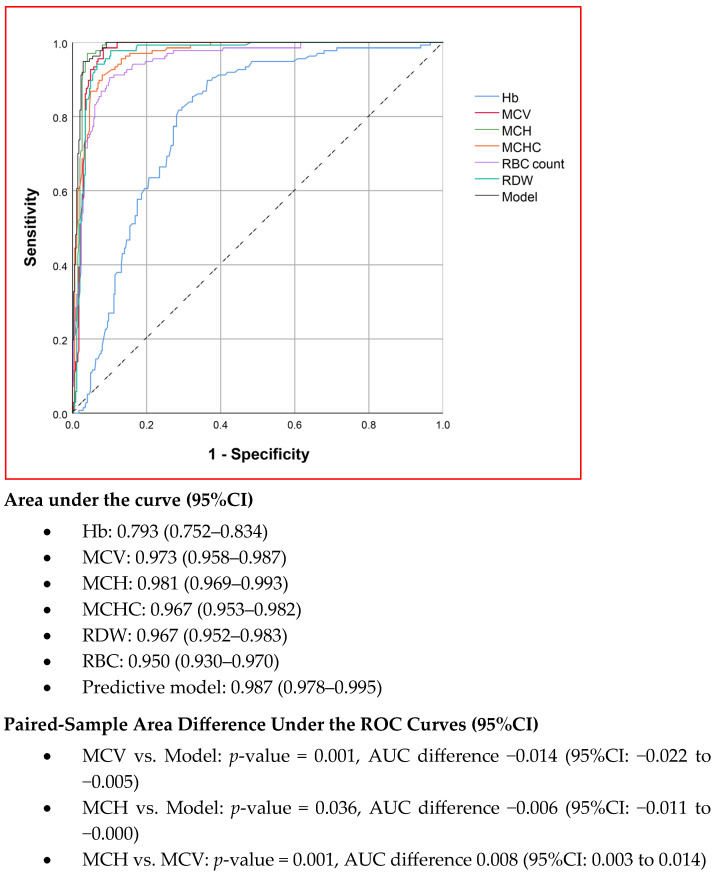
The ROC curves showing the diagnostic performance of red blood cell indices in α^0^-thalassemia trait prediction. (Hb, hemoglobin; MCV, mean corpuscular volume; MCH, mean corpuscular hemoglobin; MCHC, mean corpuscular hemoglobin concentration; RDW, red blood cell distribution width; RBC, red blood cell counts).

The diagnostic performances of MCV, MCH, MCHC, RDW, RBC count, and the combination model in predicting α^0^-thalassemia carrier status were analyzed using ROC curves, as shown in Figure 1. Among the individual parameters, MCH demonstrated the highest diagnostic performance, with an area under the curve (AUC) of 0.981, followed by MCV, which had an AUC of 0.973. The difference between these two parameters was statistically significant (*p* = 0.001, based on the paired-sample area difference test). Nevertheless, the combination model exhibited the highest performance, with an AUC of 0.987, which was significantly greater than that of both MCV and MCH.

Based on the ROC curves for MCV, MCH, and the combination model, the optimal cut-off values for defining abnormal test results were identified to maximize sensitivity while maintaining acceptable specificity: 80 fL for MCV, 26 pg for MCH, and a probability threshold of 7% for the combination model. The sensitivity, specificity, and positive likelihood ratios of these three predictors were analyzed and are presented in Table 3. Notably, all three predictors demonstrated a sensitivity of 100%, with comparable and clinically acceptable specificity, although the differences were statistically significant. The combination model achieved the highest specificity at 90.8%, while MCH exhibited higher specificity than MCV.

## 4. Discussion

The new insights gained from this study are as follows: (1) Most RBC parameters differ significantly between males with the α^0^-thalassemia trait and normal males, suggesting that these parameters are useful for distinguishing α^0^-thalassemia carriers from non-carriers. (2) Among all RBC indices, MCH demonstrated the highest diagnostic performance as a single-parameter test for predicting the α^0^-thalassemia trait, followed by MCV. Both indices exhibited a sensitivity of 100%; however, MCH had a slightly but significantly higher specificity, resulting in a lower false-positive rate. (3) The predictive model, which combines six RBC parameters, further improved screening efficiency by significantly reducing the false-positive rate. Based on these findings, MCH should be considered the first-line screening test for α^0^-thalassemia carriers. However, to maximize screening accuracy, the combined predictive model is preferable, as it significantly reduces false-positive cases and, consequently, the number of individuals requiring molecular confirmation. Notably, this improvement in diagnostic performance can be achieved without additional costs or service burdens, as all RBC parameters are routinely obtained from a standard complete blood count test.

In Southeast Asian countries, particularly Thailand, where the prevalence of α^0^-thalassemia carriers is high, there is a correspondingly high prevalence of at-risk couples and fetuses affected by Hb Bart’s disease. Consequently, a national screening policy has been implemented to identify α^0^-thalassemia carriers and facilitate early prenatal diagnosis [20,21]. In routine clinical practice, MCV is commonly used as the primary screening test, with a cut-off value of 80 fL, followed by PCR (SEA-type deletion) as the confirmatory test for both women and their partners. This approach is based on the evidence that MCV values do not differ significantly between sexes. However, this study demonstrates that other RBC parameters are also predictive of carrier status. Theoretically, incorporating multiple RBC parameters could enhance screening efficacy. However, parameters such as Hb, RDW, and RBC count differ significantly between males and females [22]. Therefore, to optimize the use of these parameters, sex-specific cut-off values and predictive models should be strongly considered.

In addition to RBC parameters, other techniques have been developed for screening α^0^-thalassemia carriers. For example, Makonkawkeyoon et al. [23] developed an ELISA strip based on a highly specific monoclonal antibody against Hb Bart’s for detecting α-thalassemia carriers, demonstrating a sensitivity of 93.4% and a specificity of 93.7%. Similarly, Tayapiwatana et al. [24] developed a sandwich-type immunochromatographic strip test for α^0^-thalassemia carrier screening, reporting a sensitivity of 100% and a specificity of 86%. However, these tests are rarely used in clinical practice due to their additional cost, limited availability, and the requirement for an extra test beyond routine laboratory assessments in standard antenatal care. Moreover, as shown in our study, their specificity is not superior to that of simple RBC parameters, further limiting their practical utility.

The RBC combination model demonstrated highly effective diagnostic performance in screening for the α^0^-thalassemia trait among males, with a sensitivity of 100% and a specificity of 90.8%. This implies that no carriers would be missed during screening while maintaining an acceptable rate of PCR testing. Obviously, the false-positive rate was considerably lower than that reported in primary care hospitals in Thailand, which ranged from 20.1% to 36.1% [25]. In practice, either MCV or MCH as a standalone parameter, or the combined predictive model, exhibited excellent sensitivity and comparable specificity. Although the predictive model demonstrated a statistically significant reduction in the false-positive rate compared to single-parameter tests, the difference may not be clinically meaningful. However, in the era of artificial intelligence, optimizing the use of simple red blood cell parameters for thalassemia risk stratification can be further enhanced through digital innovations. Notably, MCV, MCH, and MCHC are not much different between the sexes; thus, each of these parameters can be applied across both sexes within the same predictive model. However, given that hemoglobin levels and red blood cell counts exhibit significant sex-based differences, incorporating these additional parameters can further enhance predictive accuracy. Importantly, the complexity of integrating multiple variables no longer poses a limitation in the current era of advanced statistical software and artificial intelligence, which enable the seamless integration of complex models and the automated reporting of risk alongside individual parameter values from a single routine blood test. Furthermore, the incorporation of a similar model for females, which we previously developed [14], into the same application, with a sex-specific option to calculate risk according to the patient’s sex, would further enhance its applicability and clinical utility.

The weaknesses of this study include the following: (1) The diagnostic performance was sex-specific, complicating its practical application. (2) False-positive cases (abnormal parameters without the α^0^-thalassemia trait) were not further investigated to determine the underlying causes such as subclinical iron deficiency. (3) Interpretation should be approached with caution in individuals who are β- or α^+^-thalassemia carriers, as this may lead to false-positive results for the α^0^-thalassemia trait. Additionally, it should be noted that, while the model is effective, the result was obtained under the relatively controlled conditions of a research setting. In clinical practice, caution must be exercised, as several factors may affect the reliability of hematological measurements. For instance, if blood samples are left unprocessed for extended periods, cellular congestion and swelling can occur, leading to artificially elevated MCV values and potentially resulting in false-negative classifications. (4) The participants were not recruited from a purely unselected population. Several individuals were referred for prenatal diagnosis due to a known risk of having fetuses with thalassemia in previous pregnancies. Additionally, couples with a family history of the disease were more likely to undergo screening. Consequently, the prevalence of the α^0^-thalassemia trait in this study was higher than that in the general population. Nevertheless, the baseline characteristics of both groups were comparable, and participants with underlying diseases were excluded. Combined with the prospective recruitment approach, the study population likely represents non-anemic males who would typically undergo thalassemia carrier screening. Consequently, the findings are likely generalizable to the broader male population; however, external validation is required before these results can be applied in routine clinical practice.

## 5. Conclusions

The definitive diagnosis of α^0^-thalassemia carriers is crucial for identifying couples at risk of having a fetus affected by Hb Bart’s disease, particularly in regions with a high prevalence of the condition. Simple screening using RBC parameters to identify at-risk males for confirmatory PCR testing has proven to be highly effective. While most RBC parameters have strong predictive potential, the combined predictive model demonstrated a slightly but significantly lower false-positive rate, thereby reducing the need for further confirmatory testing or minimizing unnecessary molecular diagnostic costs. To enhance screening efficiency without additional effort or expense, integrating the predictive model into automated hematology analyzers could enable real-time calculation and reporting of thalassemia risk alongside RBC index values, facilitating streamlined clinical application.

## Figures and Tables

**Table 1:** Demographic data of the participants in both groups.

Characteristics	Normal Controls (N: 349)	α^0^ Thalassemia Carriers (N: 137)	*p*-Value *
Continuous variables	Mean + SD	Mean + SD	
Age (year)	30.1 ± 7.0	30.9 ± 6.8	0.268
Body weight (Kg)	67.2 ± 7.7	67.5 ± 8.5	0.694
Height (cm)	166.8 ± 13.5	167.7 ± 12.4	0.505
Body mass index (Kg/m^2^)	24.5 ± 4.8	24.4 ± 4.8	0.694
Categorical variables	N (%)	N (%)	
Nationality			0.216
Thai	341 (97.7)	131 (65.6)	
Others	8 (2.3)	6 (4.4)	
Residency			0.814
Chiang Mai	248 (71.7)	95 (69.3)	
Northern Thailand other than Chiang Mai	641 (18.5)	26 (19.0)	
Other parts of Thailand	34 (9.8)	16 (11.7)	
Education			0.514
Uneducated	20 (6.4)	10 (8.8)	
Elementary school	20 (6.4)	7 (6.2)	
Secondary school	89 (28.5)	35 (31.0)	
Diploma/vocational certificate	48 (15.4)	22 (19.5)	
Bachelor degree or higher	135 (43.3)	39 (34.5)	
Occupation			0.764
Agriculture	16 (4.6)	9 (6.7)	
Commercial	17 (4.9)	8 (5.9)	
Employee	192 (55.0)	70 (51.9)	
Government officer	44 (12.6)	13 (9.6)	
Private business	28 (8.0)	14 (10.4)	
Others	52 (14.9)	21 (15.6)	
Smoking	34 (9.7)	14 (10.2)	0.874

* Student’s *t*-test for continuous variables and Chi-square test for categorical variables.

**Table 2:** Comparisons of red blood cell indices between the α^0^-thalassemia carriers and normal controls.

Parameters	Normal Controls (N: 349)	α^0^ Thalassemia Carriers (N: 137)	*p*-Value *
Hb (g/dL)	14.9 ± 1.4	13.6 ± 0.9	<0.001
Hct (%)	44.5 ± 4.0	43.1 ± 2.9	0.222
MCV (fL)	85.6 ± 6.7	67.2 ± 3.9	<0.001
MCH (pg)	28.8 ± 2.7	20.7 ± 1.4	<0.001
MCHC (g/L)	33.7 ± 2.1	30.7 ± 0.9	<0.001
RDW (%)	12.9 ± 1.6	17.3 ± 1.8	<0.001
RBC counts (x1012/L)	5.2 ± 0.5	6.6 ± 1.4	<0.001

* Student’s *t*-test. Hb, hemoglobin; Hct, hematocrit; MCV, mean corpuscular volume; MCH, mean corpuscular hemoglobin; MCHC, mean corpuscular hemoglobin concentration; RDW, red blood cell distribution width; RBC counts, red blood cell counts.

**Table 3:** The diagnostic performance of MCV, MCH, and the combination predictive model.

Parameters	Sensitivity (95% CI)	Specificity (95% CI)	Positive Likelihood Ratio (95% CI)
Mean corpuscular volume (MCV) (Cut-off value 80 fl)	137/137: 100.0% (97.3–100.0%)	303/349: 86.8% (82.8–90.2%)	7.59 (5.8–9.9)
Mean corpuscular hemoglobin (MCH) (Cut-off value 26)	137/137: 100.0% (97.3–100.0%)	309/349: 88.5% (84.7–91.7%)	8.72 (6.5–11.7)
Combination (Predictive model) (Cut-off value 7%)	137/137: 100.0% (97.3–100.0%)	317/349: 90.8% (87.3–93.6%)	10.9 (7.8–15.2)

## Data Availability

The datasets analyzed during the current study are available from the corresponding author upon reasonable request.
